# HSV-1 progeny production by individual human keratinocytes spans three orders of magnitude

**DOI:** 10.1128/jvi.01138-25

**Published:** 2025-08-20

**Authors:** Vincent P. Villani, Jason A. Espinoza, Nir Drayman

**Affiliations:** 1 Department of Molecular Biology and Biochemistry, University of California Irvine 8788, Irvine, California, USA; 2 Department of Microbiology and Molecular Genetics, University of California Irvine 8788, Irvine, California, USA; 3 Center for Virus Research, University of California Irvine 8788, Irvine, California, USA; 4 Center for Complex Biological Systems, University of California Irvine 8788, Irvine, California, USA; University of Virginia, Charlottesville, Virginia, USA

**Keywords:** single cell, progeny production, herpes simplex, keratinocytes

## Abstract

A fundamental property of viruses is their burst size—the amount of progeny produced during one round of infection. Herpes simplex virus 1 (HSV-1) is a widespread pathogen, causing significant morbidity in the human population worldwide. Despite decades of investigation into the biology of HSV-1, its burst size at the single-cell level has not been rigorously assessed. Here, we characterized HSV-1 progeny production by individual human keratinocytes, the physiological target of HSV-1 infection. Using a fluorescently-tagged virus, high-throughput single-cell sorting, and miniaturized plaque assay, we have quantified the progeny released from over a thousand individual cells throughout infection, at two multiplicities of infection. We found that individual keratinocytes harbor a very small number of infectious viruses inside them, which necessitated physical separation of cells to account for both secreted and cell-associated progeny. The total progeny produced by individual cells spans three orders of magnitude, and this variability is observed at both high and low multiplicities of infection, with a small sub-population of super-producer cells responsible for the majority of produced progeny by the entire population. We further found that super-production is not a genetic trait of the virus, as amplification of viruses produced by low and high producer cells results in similar burst sizes. Our findings both shed light on the fundamental process of progeny production during HSV-1 infection and provide a novel target for antiviral development, aimed at blocking super-producer cells.

**IMPORTANCE:**

Viruses are defined by their ability to take over host cells and turn them into factories that produce new progeny. Recent advances in Biology allowed us to study viral infections at the single-cell level, illuminating the vast heterogeneity presented by genetically identical cells during viral infection. Despite these advances, for many viruses, we still lack even basic characterization of progeny production at the single-cell level, due to the highly demanding technical challenges associated with measuring it. Here, we present the first quantification of progeny production by individual human keratinocytes infected by herpes simplex virus 1, revealing most individual cells produce a low level of new progeny, while a few rare cells produce significantly more.

## INTRODUCTION

Perhaps the most fundamental property of viruses is their ability to reproduce. Viral progeny production is commonly studied by averaging over millions of infected cells. However, this approach overlooks the substantial heterogeneity in viral output at the single-cell level, as reported for a number of RNA viruses, including WEEV (1), Polio virus (2, 3), VSV (4, 5), and Influenza A virus (6, 7). herpes simplex virus type 1 (HSV-1) is a large double-stranded DNA virus, a major human pathogen (8) that is estimated to infect around two-thirds of the global population (9). While *in vitro* HSV-1 is capable of productive infection of multiple cell types, *in vivo* its lytic replication in the skin is mostly restricted to keratinocytes (10, 11). We and others have previously reported that individual cells infected by HSV-1 differ in their infection outcomes (12–17), viral gene expression kinetics (18–21), cellular responses (12, 13, 22, 23), and even the behavior of single viral genomes within a single infected cell (24–27). However, how this variability manifests at the progeny production level remains poorly understood. At the population level, the burst size of HSV-1 has been estimated to range from less than 100 to more than 1,000 PFU per cell, depending on the cell type, timing of measurement, and multiplicity of infection (MOI) used (28–32). A common estimate in the field is that on average, an HSV-1-infected cell produces 500–1,000 PFU. Despite decades of research into the biology of HSV-1, only a single study from 1959 investigated its progeny production at the single-cell level, by measuring the progeny produced by ~80 individual HeLa cells that were suspended in media drops under liquid paraffin, and found that under these conditions individual cells produced 1–65 new progeny (33). In a recent publication, Nobe et al. sorted sub-populations of infected cells and measured their produced progeny, finding ~10^4^ PFU were produced from 10^4^ cells, that is, an average of about 1 PFU/cell (34). However, these measurements only accounted for infectious intracellular progeny, a small fraction of produced progeny (35), as any secreted or cell-associated progeny would have been lost during the washing and trypsinization prior to sorting.

Here, we optimized the conditions required to measure the total progeny produced by individual cells and quantified progeny production from over a thousand individual human keratinocytes, uncovering the immense variability among individually infected cells, spanning three orders of magnitude.

## RESULTS

### Individual cells contain very few intra-cellular infectious progeny

**Fig 1:**
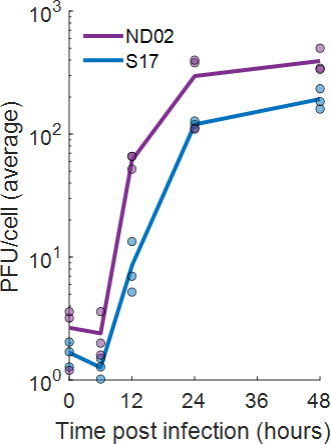
Progeny production by ND02 and the parental strain 17. Vero cells were infected by ND02 (purple) or the parental strain 17 (S17, blue) at an MOI of 10, and the total produced progeny was quantified at 0, 6, 12, 24, and 48 h post-infection by plaque assays. Lines represent the mean of three independent experiments. Individual measurements are presented by circles.

In all the experiments described here, we used the ND02 strain of HSV-1, which was derived in our lab (36) and carries two fluorescent reporters (YFP-ICP4 and RFP-VP26) that allow for easy quantification of infection by flow cytometry and microscopy. We compared the single-cycle growth curve of ND02 and the parental wild-type strain 17 (S17) and found they are similar (Fig. 1), suggesting that ND02 faithfully recapitulates progeny production kinetics.

The ND02 stock was grown and titrated on Vero cells, and the MOI was calculated based on the Vero titer. Since different cell types show different susceptibility to HSV-1 infection, we determined the percentage of infected keratinocytes at different MOIs and found that they are ~2- to 3-fold less susceptible than Vero cells (Fig. S1). Unless otherwise stated, all experiments were performed at a Vero MOI of 20, which resulted in >95% of keratinocytes becoming infected in the first round of infection (Fig. S1), to promote a synchronous infection.

**Fig 2:**
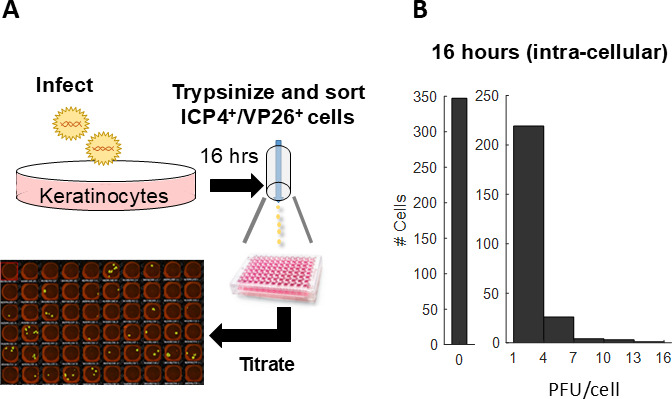
Infected keratinocytes harbor few infectious intra-cellular progeny. (A) Schematic of the experiment—human keratinocytes were infected with ND02, and ICP4^+^/VP26^+^ cells were sorted 16 h later into individual wells containing media. Cells were lysed by freezing and thawing, and the lysate was titrated by plaque assays. (B) Histograms depicting the number of infectious intra-cellular progeny for individual keratinocytes (*n* = 600 cells).

**Fig 3:**
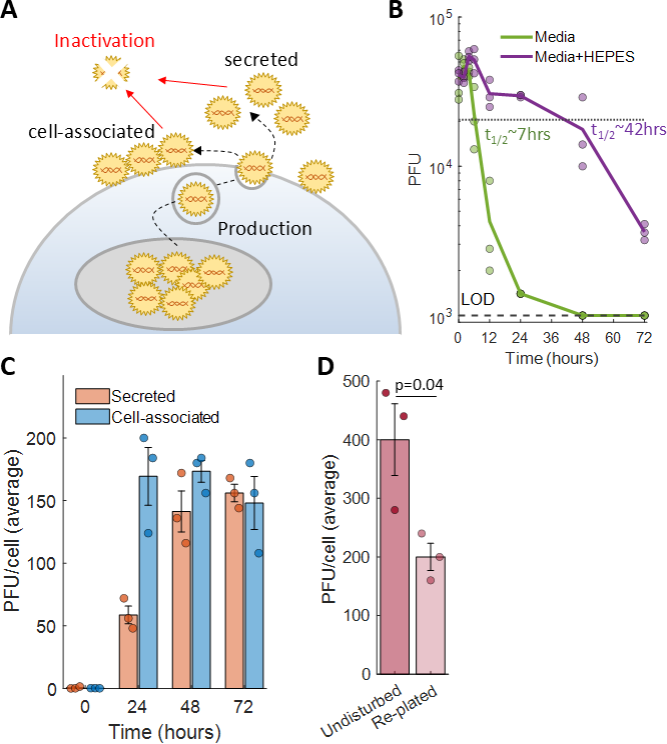
Optimized conditions for quantifying progeny production by individual keratinocytes. (A) A schematic of progeny production and inactivation during HSV-1 infection. New progeny are produced in the nucleus, travel to the plasma membrane, and are either secreted into the media or remain cell-associated, where they remain infectious until undergoing inactivation. (B) Kinetics of infectivity decay of ND02 in keratinocyte growth media either supplemented (purple) or not (green) with a pH stabilizing agent. Lines depict the mean of three independent experiments. Individual measurements are shown as circles. *t*_1/2_—estimated half-life. LOD—limit of detection. (C) Quantification of the relative proportions of secreted (orange) or cell-associated (blue) progeny during HSV-1 infection of keratinocytes. Bars and error bars depict the mean ± standard error of three independent experiments, and circles denote the individual measurements. (D) Quantification of the amount of progeny produced by infected keratinocytes left undisturbed (dark pink) or trypsinized and re-plated (light pink) immediately after viral adsorption. Bars and error bars depict the mean ± standard error of three independent experiments, and circles denote the individual measurements. *P* value was calculated by a two-tailed Student’s *t*-test.

We first characterized the amount of infectious intra-cellular progeny. Infected keratinocytes were trypsinized 16 h post-infection and individual ICP4^+^/VP26^+^ cells were sorted into 96-well plates. The cells were lysed by freeze-thawing and the amount of progeny was quantified by plaque assays (Fig. 2A). As trypsin inactivates cell-associated HSV-1 (37), and since any secreted progeny is not accounted for, these measurements only capture infectious intra-cellular progeny, that is, viruses that have acquired their final envelope and are en route to be secreted (Fig. 3A) (35). Our measurements indicate that individual cells contain very few infectious progeny, ranging from 0 to 15 PFU/cell (Fig. 2B), with an average of 2.7 PFU/cell (Table 1). These results are in agreement with recently reported bulk measurements in several cell types, showing intra-cellular infectious progeny averages ~1 PFU/cell (34). Thus, while we can clearly observe variability among individual cells, this approach misses the majority of produced progeny.

**TABLE 1:** Summary of statistics for progeny production by individual cells

Parameter	MOI 20				MOI 1 48 h
	16 h (intracellular)	24 h	48 h	72 h	
Number of cells	253	312	328	418	113
Mean	2.7	32	62	20	26
Standard deviation	1.9	48	77	17	46
Median	1.7	14	38	16	14
Gini coefficient	0.29	0.61	0.56	0.43	0.61
% progeny from top 10% producers	20%	43%	40%	28%	45%
% progeny from top 25% producers	33%	72%	65%	53%	70%

### Optimizing conditions for measuring progeny production from individual cells

As the amount of infectious virus present at any given time is the sum of progeny production and virus inactivation (Fig. 3A), we set to minimize viral inactivation in our system. We determined the kinetics of HSV-1 inactivation in the keratinocyte growth media under cell growth conditions (37°C, 5% CO_2_, and 100% humidity) and found that under these conditions, HSV-1 infectivity decays rapidly, with a half-life of ~7 h and over 90% inactivation by 12 h (Fig. 3B). This decay is mostly pH dependent, as supplementing the growth media with 25 mM HEPES to maintain the pH ~7.4 was sufficient to significantly improve HSV-1 stability, increasing the half-life to ~42 h (Fig. 3B). We thus used the pH-stabilized media in all subsequent experiments, allowing us to minimize the impact of viral inactivation on progeny production measurements.

We next determined which route is used by human keratinocytes to release new progeny, as HSV-1 is known to spread either through secretion by exocytosis or through direct cell-to-cell contact (38–41). We infected cells and quantified the amount of progeny in the secreted and cell-associated fractions and found that the majority of progeny were cell-associated during the first 24 h after infection, and about equal amounts were cell-associated or secreted thereafter (Fig. 3C). The burst size of ND02 in infected keratinocytes at 48 h averaged 314 PFU/cell (141 secreted + 173 cell-associated, Fig. 3C), similar to that determined in Vero cells above (393 PFU/cell, Fig. 1).

Thus, to properly quantify progeny production from individual keratinocytes, both cell-associated and secreted viruses must be quantified. Quantifying both fractions from individual cells presents a logistical problem as cells must be physically separated to prevent mixing of their produced progeny in the media. We therefore tested whether cells trypsinized and re-plated immediately after viral adsorption go on to reattach and produce progeny. We infected cells and either left them undisturbed or trypsinized and re-plated them and quantified the amount of total progeny (secreted + cell-associated) produced 48 h later. We found that infected keratinocytes are indeed able to reattach and produce progeny, albeit at a reduced capacity, with the average burst size decreasing by half (Fig. 3D).

### Individual keratinocytes exhibit high variability in the amount of progeny they produce

**Fig 4:**
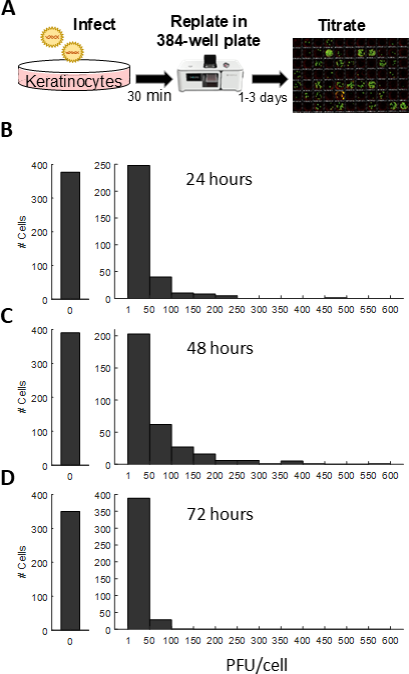
HSV-1 progeny production by individual keratinocytes. (A) A schematic representation of the experimental pipeline: keratinocytes were infected in bulk at an MOI of 20, trypsinized and re-plated as individual cells in 384-well plates containing media. The cells were lysed by freezing and thawing at 24, 48, and 72 h post-infection, and the total progeny produced by each cell was quantified by plaque assays. (B–D) Histograms depicting the amount of progeny produced by individual cells at different time points (*n* = 2,175 cells). The experiment was performed two times for each time point, and the aggregated results are shown. See Fig. S2 for the results of the individual repeats.

Using these optimized conditions, we set out to measure the amount of progeny produced by individual keratinocytes. We infected cells, trypsinized them immediately after viral adsorption, and sorted individual cells into 384 well plates (Fig. 4A). To minimize the effect of sorting on cell viability and progeny production, we used a low-pressure single-cell sorter (NX1, Nodexus). The sorter was located in our lab, such that time spent outside of a cell incubator was limited to ~30 minutes. Under these sorting conditions, uninfected keratinocytes grew into single colonies in 60% of sorted wells, with a low rate of doublets (2%).

Plates containing single infected cells were then incubated for 24–72 h, and the amount of total produced progeny was determined by titrating each well’s content using the plaque assay. Two independent measurements were performed for each time point, for a total of 2,175 cells across all conditions (see Fig. S2 for results of the individual repeats).

The kinetics of progeny production at the single-cell level mirrored that of cells re-plated in bulk, peaking at 48 h post-infection (Fig. S3). Given the half-life of the virus in the media, it is likely that no new progeny were produced after 48 h.

At 48 h post-infection, progeny production ranged from 3 to 600 PFU/cell (Fig. 4C), with a median of 38 and an average of 62 (Table 1), about threefold less than the average burst size observed for re-plated cells in bulk (Fig. 3D). Most of the progeny were contributed by a small group of super-producers, with the top 10% of cells accounting for 40% of the total progeny. Table 1 summarizes the statistics for progeny production by individual cells at the different time points.

A significant portion of the assayed single cells did not produce detectable progeny (~50%, Fig. 4B through D), on par with previous reports for Polio virus (3) and Influenza A virus (6, 7), which can be explained by either *bona fide* abortive infections (infected cells that do not produce new progeny) or low cell viability following single-cell sorting. We quantified the percentage of abortive infections by infecting keratinocytes as above, incubating the infected cells for 3 h followed by trypsinization and sorting of individual YFP-ICP4^+^ cells onto 96-well plates containing monolayers of uninfected keratinocytes. We incubated the plates for 5 days and scored each well as abortive (no detected HSV-1 spread) or productive (detected HSV-1 spread). This assay allows for sensitive discrimination between abortive and productive infections, as any progeny produced by the initially sorted cell is amplified. We found that under these conditions, 73% of cells that express ICP4 go on to produce progeny (Fig. S4), while 27% do not. Taken together with the fact that only 60% of uninfected sorted cells are able to grow and form colonies, we conclude that both cell viability and abortive infections contribute to the large proportion of single cells with no detectable progeny production (Fig. 4).

### MOI does not explain cell-to-cell variability in progeny production

We hypothesized that the variability in progeny production by single cells might be explained by the number of infecting viruses. To test this, we repeated the measurements performed above at a Vero MOI of 1 (38% infected keratinocytes, Fig. S1), enumerating the number of progeny produced 48 h post-infection (Fig. S5; Table 1). Our results indicate that cells infected at a lower MOI produced less progeny on average (average of 26 PFU/cell for MOI = 1 vs 62 PFU/cell for MOI = 20, Table 1), but the overall cell-to-cell variability was not impacted, with the amount of progeny produced by single cells ranging from 1 to 267, and comparable Gini coefficients and fraction of super-producer cells (Table 1). Thus, while the number of infecting virions is important in setting the averaged progeny production, it does not directly impact the cell-to-cell variability in progeny production.

### Super-production is not an inheritable viral trait

**Fig 5:**
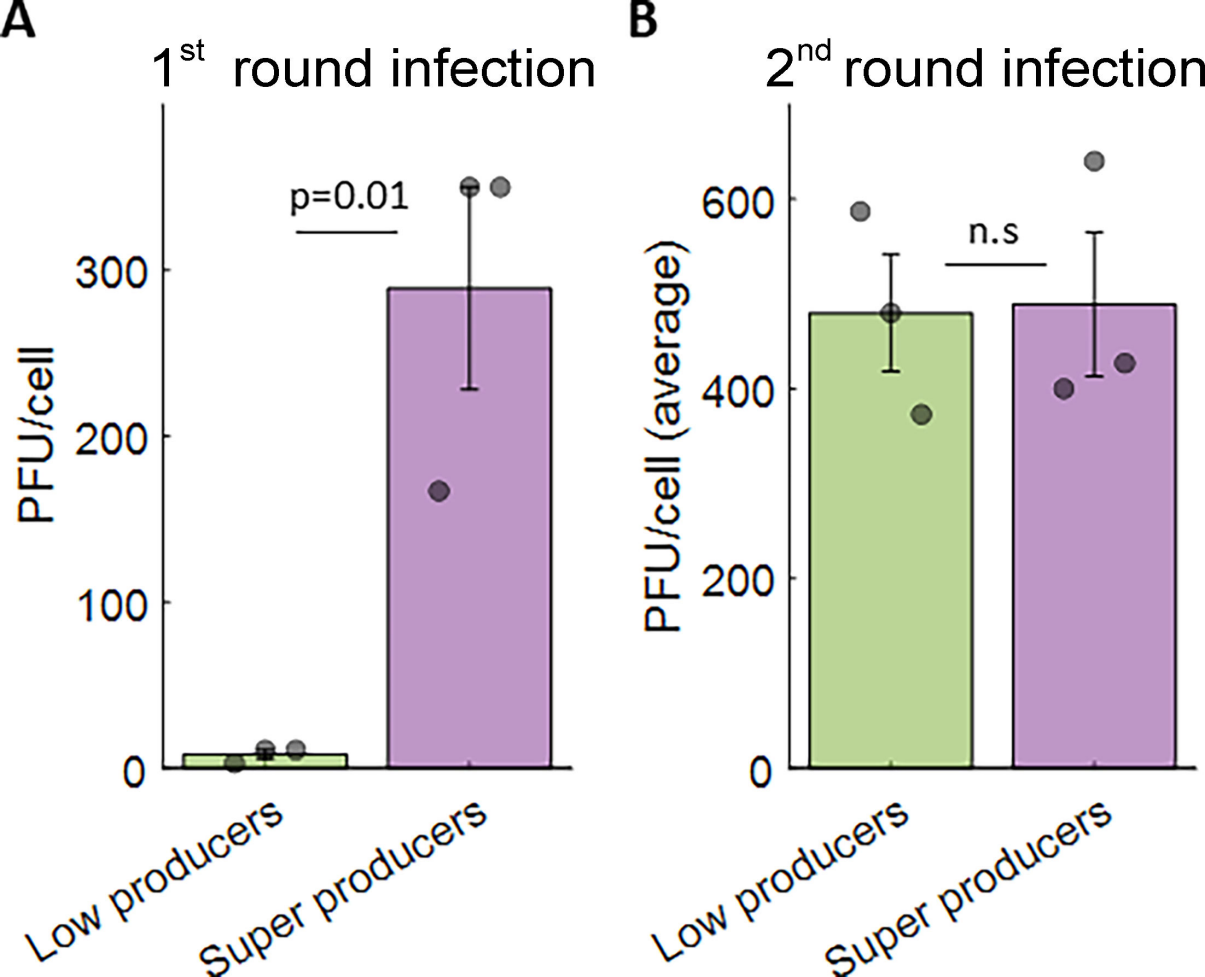
Super-production is not an inherited viral trait. (A) initial titers from three low producer cells (green) and three super producer cells (purple). (B) Burst sizes of the same viruses after one round of amplification and infection of keratinocytes at an MOI of 20 for 48 h. Bars and error bars depict the mean ± standard error of three independent measurements, and circles denote the individual measurements. *P* values were calculated by a two-tailed Student’s *t*-test.

Since the MOI did not explain super-production, we wondered if genetic differences in the viral population might. To test this, we amplified the progeny from both low and super producers, titrated the resulting stocks on Vero cells, and determined the average burst size by infecting keratinocytes at an MOI of 20, collecting total progeny (secreted and cell-associated) 48 h later and determining the amount of progeny by plaque assay (Fig. 5). Our results indicated that progeny of both low and super producer cells have similar burst sizes, excluding genetic variability in the viral population as the source of cell-to-cell variability in progeny production during HSV-1 infection.

In summary, we present here the first measurement of HSV-1 progeny production by individual human keratinocytes. We find that progeny production by individual cells is highly heterogeneous, spanning three orders of magnitude, as well as highly unequal, with a small group of super-producers responsible for most of the progeny production by the entire population. While the factors controlling this phenomenon are yet unknown, we have ruled out two likely factors - the MOI and viral genetics.

## DISCUSSION

Here, we analyzed HSV-1 progeny production by human keratinocytes at the individual cell level. We found that the average burst size of ND02 is ~400 PFU/cell and is similar when infecting Vero cells (Fig. 1) or human keratinocytes (Fig. 3). We showed that individual infected keratinocytes harbor very few infectious intra-cellular progeny (Fig. 2) and that to properly account for progeny production both secreted and cell-associated progeny need to be collected (Fig. 3). Using a low-pressure single-cell sorter, we were able to seed individually infected keratinocytes in 384-well plates and quantify the amount of progeny each cell produces, showing it spans three logs in variability and that a small subset of super producers is responsible for the majority of produced progeny (Fig. 4; Table 1). Investigating potential sources of this variability, we have ruled out a role for MOI (Table 1) and viral genetics (Fig. 5) in super-production.

There are several limitations to our measurements. First, while unavoidable, the necessity to physically separate single cells creates a non-physiological scenario, as the cellular microenvironment is an important factor in viral infection (20, 42). The isolation of single cells in wells, either due to the lack of neighbor cells or as a byproduct of sorting, has a measurable impact on progeny production, as the average PFU/cell in our single-cell measurements at 48 h (62 PFU/cell, Table 1) is ~3-fold lower than for cells re-plated in bulk (200 PFU/cell, Fig. 3D). Thus, our measurements likely underestimate the number of progeny produced by individual cells, but not the heterogeneity among them. Second, the described experiments are limited to a single viral recombinant, ND02. While we have shown that ND02 has similar replication kinetics to its parental strain 17 (Fig. 1), how other HSV-1 strains behave remains an open question. Finally, our studies have been conducted in a single monoclonal immortalized keratinocyte cell line (N/TERT-670), which is expected to recapitulate basal keratinocyte behavior, but not the other cell types present in the human skin (43).

A key unanswered question is what determines the amount of progeny produced by individual cells. By necessity, burst size measurements at the cell population level are performed at a high MOI (28–32), since lower MOIs result in secondary infections that make results hard to interpret. Using our single-cell approach, we compared progeny production at MOIs of 1 and 20 and found that a lower MOI infection resulted in a lower average PFU/cell (Table 1), in agreement with a previous report from the Kobiler lab, showing that the magnitude of viral gene expression in single cells is correlated with the number of replicating genomes in the nucleus (18). However, while the MOI significantly impacted overall progeny production, it did not significantly affect the heterogeneity among individual cells as measured by the Gini coefficient or the fraction of super-producers (Table 1), suggesting other factors contribute to cell-to-cell variability in progeny production. In all likelihood, both cell-to-cell and virus-to-virus variability contribute to this heterogeneity. While we have ruled out viral genetic variability as the source of super-producer (Fig. 5), many other non-genetic differences could be the source. For example, Pietilä et al. have recently described how cellular states are connected to differences in HSV-1 transcriptional progression at the single-cell level (22) and the Lippé group has quantified the proteomic heterogeneity between individual HSV-1 particles (44, 45). Another intriguing possibility is that virion infectivity, rather than the total number of virions, is different between progeny produced in individual cells. HSV-1 infection is known to result in many defective or non-infectious progeny (12, 46, 47), and a recent CRISPR screen has shown that some host factors modulate virion infectivity for human cytomegalovirus (48). While we could not measure the genomes to PFU ratio in our single-cell pipeline due to technical reasons, owing to the miniscule amount of materials, future studies could address this possibility. While far from trivial, future studies should aim to identify which host and viral factors play a role in determining which cells become super producers, as such factors could be important targets for novel therapies.

## MATERIALS AND METHODS

### Cell lines and viruses

N/TERT-2G cells, immortalized human keratinocytes, were described by Dickson et al. (49) and generously provided by Dr. Scott Atwood from the University of California, Irvine. The N/TERT-670 cell line was derived from the N/TERT-2G by our lab, through lentiviral transduction of a transgene expressing H2B-miRFP670, a far-red fluorescent protein, fused to histone 2B. N/TERT-670 cells were used for all experiments described in the manuscript, except for plaque assays. The cells were maintained in keratinocyte serum-free media (KSFM) supplemented with human recombinant epidermal growth factor, bovine pituitary extract (Gibco, catalog no. 317005042) and 1× penicillin-streptomycin (Corning 30-002). Unless otherwise stated, the media was further supplemented with 25 mM HEPES (4-(2-hydroxyethyl)piperazine-1-ethane-sulfonic acid, Sigma-Aldrich catalog no. 7365-45-9) to maintain the pH at 7.4.

Vero cells (African Green Monkey kidney cells, ATCC CCL-81) were used for growing viral stocks and plaque assays. The cells were maintained in Dulbecco’s modified Eagle medium (DMEM, Gibco, catalog no. 10566016) supplemented with 10% bovine calf serum (BCS, Cytiva, catalog no. SH30072.03) and 1× penicillin-streptomycin (Corning 30-002). The cells were grown in incubators maintaining 37°C, 5% CO_2_ and 100% humidity.

All experiments were performed with the ND02 strain of HSV-1 previously developed in our lab (36). ND02 was derived from a non-syncytial strain 17 background and expresses YFP-ICP4 and RFP-VP26 fusion proteins, allowing for fluorescent-based measurements of plaques and infected cells proportions.

### Virion inactivation kinetics

40,000 PFU of ND02 were placed in keratinocyte growth media supplemented with 25 mM HEPES or vehicle. Virions were incubated in the same incubators used to grow cells (37°C, 5% CO_2_, and 100% humidity) and aliquots were retrieved at 0–72 h and kept at −80°C until analysis. After the collection of all time points, viral titers were determined by plaque assay on monolayers of Vero cells. The half-life of virus infectivity was estimated from the curves.

### Plaque assay

Vero cell monolayers were infected with serial dilutions of samples for 30 min. Following adsorption, the inoculum was aspirated and an overlay media (DMEM + 10% BCS + 2% cellulose) was applied to the cells. The cells were incubated for 48–72 h before imaging using a Nikon Eclipse Ti2 epi-fluorescent microscope. Images of the brightfield, YFP, and RFP channels were captured using a 2× objective to capture the entire well. Plaques were manually counted using the fluorescent channels.

### HSV-1 infection

N/TERT-670 cells were seeded at 5 × 10^5^ cells per well in six-well plates the day before infection. The following day, the cells were counted and infected with ND02 at the indicated MOIs. MOIs were calculated based on titration in Vero cells. The virus was allowed to adsorb to cells for 30 min in a total volume of 200 µL. Plates were gently shaken after 15 min to prevent cells from drying. Following adsorption, the inoculum was aspirated, the cells were washed with KSFM, and fresh media were added. This was considered time 0 for all experiments. The cells were incubated at 37°C, 5% CO_2_, and 100% humidity until analysis.

### Measuring secreted and cell-associated fractions

N/TERT-670 cells were infected and incubated for 0–72 h. At the indicated time points, the cell supernatant, designated the secreted fraction, was collected and kept at −80°C until analysis. Fresh media were added to the cells, and the plate was stored at −80°C until analysis. One round of the freeze-thaw cycle was used to liberate progeny from the cells, designated the cell-associated fraction. We employed one round of freeze-thaw instead of the commonly used three cycles, as in our hands, the additional cycles did not result in increased progeny release, and sometimes a decrease was observed. The amount of progeny in each fraction was measured by plaque assay on Vero cells.

### Measuring the effect of cells re-plating on progeny production

N/TERT-670 cells were infected and then trypsinized for 5 min immediately after viral adsorption or left undisturbed. Trypsin was quenched with PBS + 2% BCS, and the cells were pelleted by centrifugation at 500 × *g* for 5 min. The cell pellet was resuspended, and 125,000 cells were re-plated in six-well plates. Plates were incubated for 48 h, freeze-thawed once, and the amount of total progeny (secreted + cell-associated) measured by plaque assay.

### Progeny production by single cells

N/TERT-670 cells were seeded at 5 × 10^5^ cells per well in six-well dishes and incubated overnight at 37°C. The following day, the cells were infected for 30 min, washed to remove residual virus, and then detached with trypsin for 5 min at 37°C. The reaction was quenched with PBS + 2% BCS, and the cells were pelleted by centrifugation for 5 min at 500 × *g*. The cell pellet was re-suspended in KSFM and individual cells were sorted into 384-well plates containing 50 µL of KSFM supplemented with 25 mM HEPES, using the low-pressure Nodexus NX-01 single-cell sorter, located in our lab. The cells were kept at room temperature during sorting. The entire process from trypsinization to plating lasted about 30 min. Plates were spun down at 500 × *g* for 5 min to encourage cell attachment and incubated for 24–72 h. Plates were then freeze-thawed once at −80°C and 37°C, respectively, for 20–30 min each. 30 µL of the resulting cell lysates was used to infect Vero cell monolayers in 96-well plates for 30 min. The inoculum was aspirated, monolayers were washed once, and overlay media were added. Plates were incubated at 37°C for 48 h and imaged to quantify the number of plaques. Most wells contained a quantifiable number of plaques, but some were too numerous to quantify. For these wells, we repeated the titration using a 10-fold dilution from the remaining 20 µL of cell lysate.

### Software and statistical analysis

Matlab R2024b was used for data visualizations and statistics. Two-tailed Student’s *t*-test was used for two-group comparisons. FCSExpress was used for flow cytometry analysis. Nikon’s Elements was used for microscopy analysis.

## Data Availability

All data are available in the main text and supplemental material.

## SUPPLEMENTAL MATERIAL

Supplemental figures jvi.01138-25-s0001.pdf Fig. S1 to S5.

ASM does not own the copyrights to Supplemental Material that may be linked to, or accessed through, an article. The authors have granted ASM a non-exclusive, world-wide license to publish the Supplemental Material files. Please contact the corresponding author directly for reuse.
