# Supplementary material for: HSV-1 progeny production by individual human keratinocytes spans three orders of magnitude
Source: J Virol. 2025 Aug 20;99(9):e01138-25. doi: 10.1128/jvi.01138-25 (PMC12455958; doi:10.1128/jvi.01138-25)
Supplement: Supplemental figures — Fig. S1 to S5. [file jvi.01138-25-s0001.pdf]

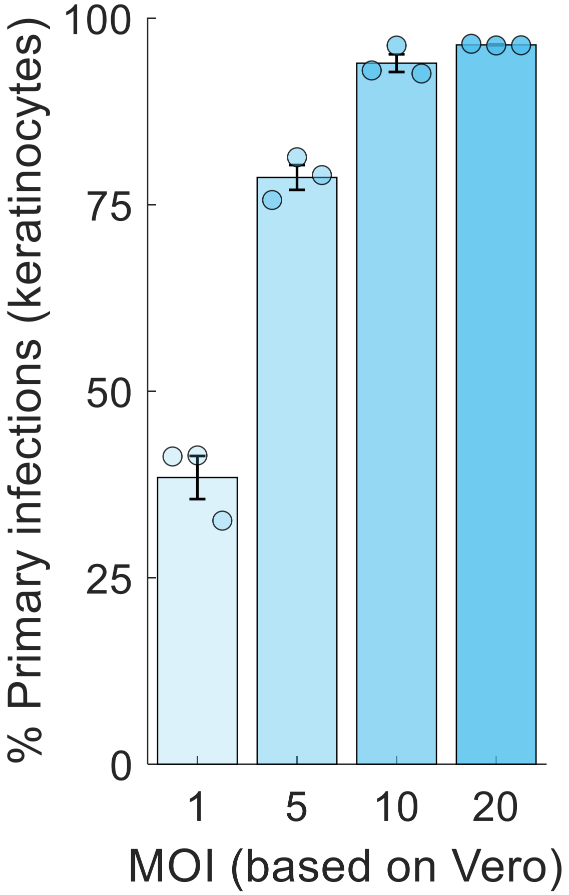

Fig. S1. Percent of primary infections as a function of MOI. Keratinocytes were infected with ND02 at the indicated MOIs (based on Vero cells), and incubated for 24 hours in the presence of 400µg/ml of PAA, an inhibitor of viral DNA replication. Cells were assayed by flow-cytometry and the percentage of primary infected cells was determined as the fraction of cells expressing YFP-ICP4. Bars and error bars depict the mean±standard error of three independent experiments and circles denote the individual measurements.

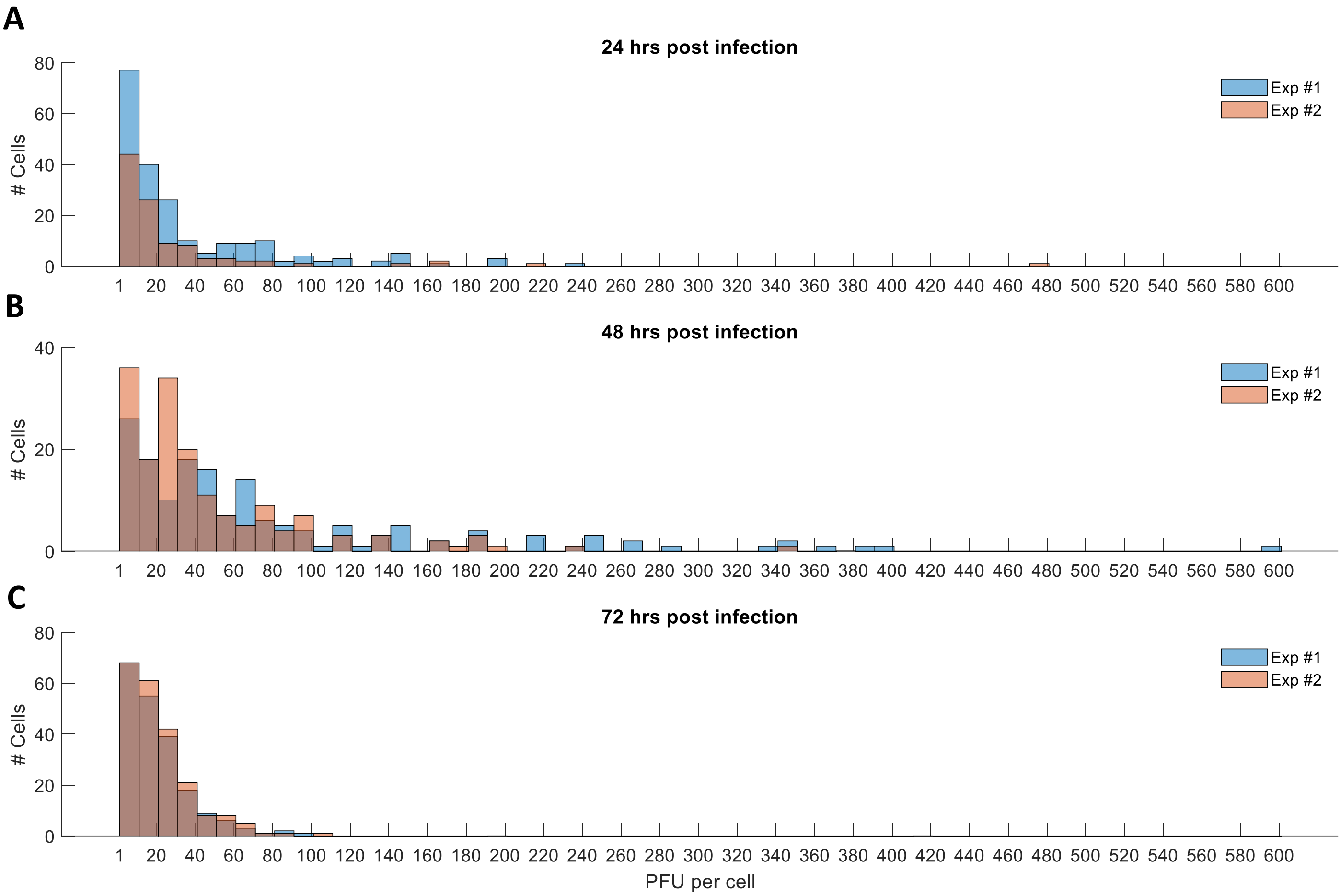

Fig. S2. HSV-1 progeny production by individual keratinocytes – individual repeats. Shown are the histograms for cells production progeny (excluding the cells with zero counts) for each of the two repeats that are presented in aggregate in Fig. 4. Repeat #1 is color coded blue and repeat #2 is orange. Note that bars are semi-transparent so where they overlap a third color is seen.

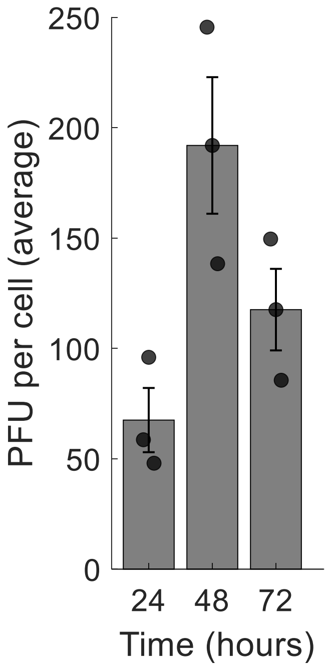

Fig. S3. Average progeny production under the same conditions used for single cells. Cells were infected as for the single cell experiment (Fig. 4) but were re-plated in bulk (125,000 cells per well in a 6-well plate). Plates were freeze-thawed at the indicated time points and total progeny assayed by plaque assay. Results are shown as average PFU per cell. Bars and error bars depict the mean±standard error of three independent experiments and circles denote the individual measurements.

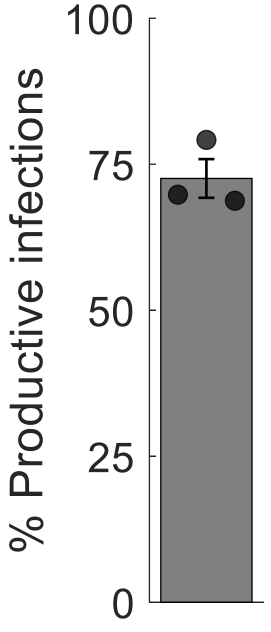

Fig. S4. Percent of productive infections. Keratinocytes were infected with ND02 at an MOI of 20, incubated for 3 hours and individual YFP-ICP4+ cells were sorted into 96-well plates containing monolayers of uninfected keratinocytes. 5 days later the wells were scored as abortive or productive based on the absence or presence of HSV-1 spread, respectively. Bars and error bars depict the mean±standard error of three independent experiments and circles denote the individual measurements.

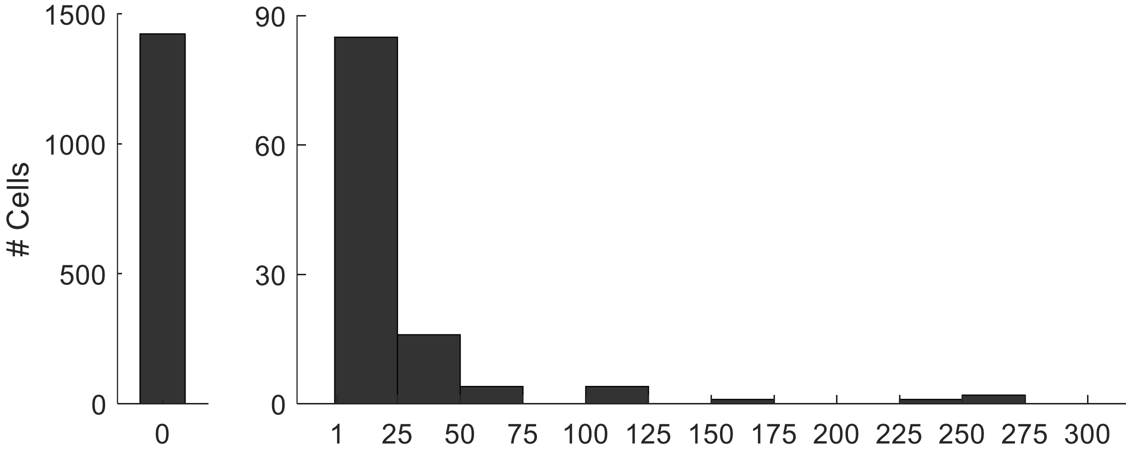

Figure S5. HSV-1 progeny production by individual keratinocytes at an MOI=1. Keratinocytes were infected in bulk at an MOI of 1, trypsinized and re-plated as individual cells in 384-well plates containing media. Cells were lysed by freezing and thawing at 48 hours post infection and the total progeny produced by each cell was quantified by plaque assays. Histograms depicting the amount of progeny produced by individual cells (n=1,536 cells). The experiment was performed twice using two 384 well plates and the aggregated results are shown.
